# Metabolic profiling detects early effects of environmental and lifestyle exposure to cadmium in a human population

**DOI:** 10.1186/1741-7015-10-61

**Published:** 2012-06-19

**Authors:** James K Ellis, Toby J Athersuch, Laura DK Thomas, Friederike Teichert, Miriam Pérez-Trujillo, Claus Svendsen, David J Spurgeon, Rajinder Singh, Lars Järup, Jacob G Bundy, Hector C Keun

**Affiliations:** 1 Biomolecular Medicine, Department of Surgery and Cancer, Faculty of Medicine, Imperial College London, Sir Alexander Fleming Building, South Kensington, London, SW7 2AZ, UK; 2 MRC-HPA Centre for Environment and Health, Imperial College London, W2 1PG, UK; 3 Department of Epidemiology and Biostatistics, School of Public Health, Faculty of Medicine, Imperial College London, London, W2 1PG, UK; 4 Unit of Nutritional Epidemiology, Institute of Environmental Medicine, Karolinska Institutet, Stockholm, Sweden; 5 Cancer Biomarkers and Prevention Group, Department of Cancer Studies and Molecular Medicine, University of Leicester, Leicester, LE2 7LX, UK; 6 Centre for Ecology and Hydrology, Maclean Building, Benson Lane, Crowmarsh Gifford, Wallingford, Oxon, OX10 8BB, UK; 7 Servei de Ressonància Magnètica Nuclear, SeRMN, Universitat Autònoma de Barcelona, Barcelona, Spain

**Keywords:** metabonomics, cadmium, environmental health, exposome, metabolomics, molecular epidemiology

## Abstract

**Background:**

The 'exposome' represents the accumulation of all environmental exposures across a lifetime. Top-down strategies are required to assess something this comprehensive, and could transform our understanding of how environmental factors affect human health. Metabolic profiling (metabonomics/metabolomics) defines an individual's metabolic phenotype, which is influenced by genotype, diet, lifestyle, health and xenobiotic exposure, and could also reveal intermediate biomarkers for disease risk that reflect adaptive response to exposure. We investigated changes in metabolism in volunteers living near a point source of environmental pollution: a closed zinc smelter with associated elevated levels of environmental cadmium.

**Methods:**

High-resolution ^1^H NMR spectroscopy (metabonomics) was used to acquire urinary metabolic profiles from 178 human volunteers. The spectral data were subjected to multivariate and univariate analysis to identify metabolites that were correlated with lifestyle or biological factors. Urinary levels of 8-oxo-deoxyguanosine were also measured, using mass spectrometry, as a marker of systemic oxidative stress.

**Results:**

Six urinary metabolites, either associated with mitochondrial metabolism (citrate, 3-hydroxyisovalerate, 4-deoxy-erythronic acid) or one-carbon metabolism (dimethylglycine, creatinine, creatine), were associated with cadmium exposure. In particular, citrate levels retained a significant correlation to urinary cadmium and smoking status after controlling for age and sex. Oxidative stress (as determined by urinary 8-oxo-deoxyguanosine levels) was elevated in individuals with high cadmium exposure, supporting the hypothesis that heavy metal accumulation was causing mitochondrial dysfunction.

**Conclusions:**

This study shows evidence that an NMR-based metabolic profiling study in an uncontrolled human population is capable of identifying intermediate biomarkers of response to toxicants at true environmental concentrations, paving the way for exposome research.

## Background

From the point of conception and throughout life, humans experience a broad range of physical, chemical and biological exposures. The health effects of such exposures will depend not only on dose but also on their interaction with each other and with the characteristics of the individual, such as age, sex and genotype. Hence, it is a persistent and significant challenge to understand how specific environmental factors produce effects on human health. Biomarkers already play an important role in characterizing both dose and effect; however, their full potential remains to be explored. Molecular profiling technologies ('-omics') have been suggested to be an important route to the discovery of novel biomarkers to improve exposure assessment in man [1]. Such techniques can also report on the biological consequences of exposure by identifying intermediate biomarkers that both correlate with exposure and predict health endpoints, termed a 'meet-in-the-middle' approach [2,3]. Such markers would help to define the mechanism of toxicity and the etiology of disease in the human population and ultimately could inform follow-up surveillance for communities impacted by environmental pollution. It has been proposed that by combining a wide range of molecular profile data, including parallel assays of molecular adducts, metals and other exogenous species, we have the potential to define the molecular imprint of the totality of environmental exposures in an individual's lifetime, the 'exposome'[4,5].

Metabolic profiling allows the study of an individual's metabolic phenotype [6,7] and represents a systematic and efficient route to intermediate biomarkers. It can detect and classify the consequences of toxicant exposure *in vivo *[8,9] and the approach readily translates to molecular epidemiology [10]. Here we use an NMR-based metabolic profiling approach to investigate changes in human systemic metabolism from a sample of the population living near a point source of environmental pollution. The site (Avonmouth, UK) was home to one of the world's largest smelters and large amounts of cadmium (Cd) and other potentially toxic elements were released into the local environment until its closure in early 2003 resulting in the presence of elevated concentrations of metals in air, soil and house dust in the area [11].

Cd is a toxic heavy metal of occupational and environmental concern due to its widespread contamination of sites worldwide [12,13] and long biological half-life (10 to 30 years) [14]. Cd exposure is associated with a host of adverse effects [15], including osteoporosis, pulmonary dysfunction, hypertension and nephropathy. The kidney accumulates Cd and is the critical organ, particularly at environmental levels of exposure, with tubular proteinuria being an early effect of Cd exposure [16]. This proteinuria is usually detected as an increased excretion of low-molecular weight urinary proteins, such as N-acetyl-β-D-glucosaminidase (NAG). There is some evidence to suggest that, following high and/or prolonged exposure, these pre-clinical changes may progress to renal impairment with a decreased glomerular filtration rate [17-19], and eventually to renal failure [20].

A further concern is cancer risk; Cd is a multi-site carcinogen in both rodents [21] and humans [22] and as such has been classified as a human carcinogen by the International Agency for Research on Cancer [23] and the National Toxicology Program [24]. The mechanism of Cd carcinogenesis is thought to be multi-factorial but is as yet poorly understood. Cd has been shown to possess estrogenic activity [25-27], inhibit mismatch repair [28], alter DNA methylation [29,30] and increase reactive oxygen species (ROS) production [31].

The diet is the major source of Cd exposure in the general non-smoking population with around 5% to 10% [32] absorption from the gastrointestinal tract. However, among smokers tobacco is also a significant source of exposure owing to the high rate of absorption from the lungs (10 to 50%) [32]. The low excretion rate of Cd and the resulting long biological half life means that Cd body burden increases slowly with age and appears to accumulate at a higher rate in women compared to men. Urinary-Cd (U-Cd) concentration is mainly influenced by the total body burden and is, hence, a marker for long term exposure, but can also increase as kidney damage occurs [14].

**Figure 1:**
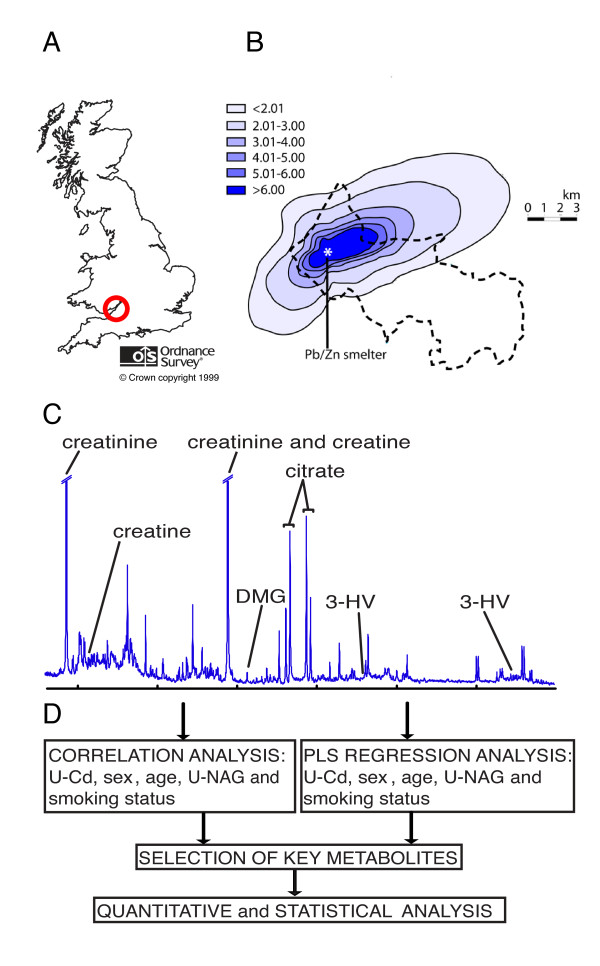
**Metabolic profiling in molecular epidemiology: a methodology to identify intermediate biomarkers of response to environmental toxicants**. **A**) Map of United Kingdom showing Bristol region. Reproduced from Ordnance Survey map data by permission of the Ordnance Survey ^© ^Crown copyright 2010. **B**) Air Cd concentration around the Avonmouth zinc smelter site (data from Thomas *et al. *(2009)[37]). Contours represent modeled air Cd concentration (ng/m^3^). Dotted line represents boundary of Bristol North PCT from where volunteers were recruited. **C**) Partial ^1^H NMR one dimensional spectrum with key metabolites annotated. 3-HV, 3-hydroxyisovalerate; DMG, dimethylglycine. **D**) Outline of the metabolic profiling and statistical methods used to define associations between the metabolome and environmental toxicants.

It has been well documented that ^1^H NMR-based metabolic profiling (metabonomics/metabolomics) can detect Cd-induced perturbations of metabolism in animal models [33-35] with renal damage and biochemical changes at concentrations that were considered relatively safe in humans by the World Health Organisation [36]. However, there have not been any comparable investigations in human populations. In the present study, we aim to establish that NMR-based metabolic profiling could be used in molecular epidemiology to define the metabolic signatures of exposure to toxicants in the human population and link these to mechanisms of toxicity. We correlated urinary metabolic profiles of individuals living near the Avonmouth site with environmental toxicants (U-Cd) and corrected for confounding lifestyle and biological factors, such as age, sex and smoking status (Figure 1).

## Methods

### Sample collection and epidemiology data

One hundred and eighty adult participants were recruited to the current study as described in Thomas *et al. *(2009) [37], and 178 samples in total were analyzed by ^1^H NMR spectroscopy. Briefly, each individual was asked to provide one spot morning urine sample; each sample was frozen on the day of collection. Each volunteer completed a lifestyle questionnaire that provided information on a number of parameters including smoking status (current, past, never), age and sex. Data were anonymized for the present study. U-Cd and U-NAG were previously determined [37] and were both log-normally distributed. Ethical approval for the study was granted by Bristol (UK) South and Central Ethics Committee and all participants provided their written informed consent before data collection.

### NMR data acquisition

Samples were thawed on ice and prepared in a single batch. For each sample, an aliquot (0.4 mL) was mixed with buffer (0.2 mL, 0.2 M sodium phosphate, pH 7.4) containing ^2^H_2_O (20% v/v) and sodium 3-trimethylsilyl-1-[2, 2, 3, 3,-^2^H_4_] propionate (TSP, 0.3 mM) and centrifuged (16,000 g, ten minutes) to remove suspended particulate matter.

High-resolution ^1^H NMR spectroscopy experiments were conducted using a Bruker AVANCE 600 spectrometer (Bruker Biospin, Rheinstetten, Germany) fitted with a flow-injection mode probehead (5 mm FI TXB ^1^H-^13^C/^15^N-^2^H Z-GRD H8432/K0201 Z8432/0201, Bruker) at a field strength of 14.1 T (600.13 MHz ^1^H frequency). The probe temperature was set to 300 K. Samples were delivered to the probehead in 96-well plates using an automated flow injection system (Bruker). Following introduction to the probe, samples were left to equilibrate (three minutes) prior to gradient shimming using the ^1^H channel to ensure good magnetic field homogeneity. All liquid sample handling, automation and acquisition were controlled using XWIN-NMR 3.1 software (Bruker) running on a UNIX workstation (Silicon Graphics, USA).

One-dimensional NMR spectra were acquired using a standard pulse sequence using excitation sculpting with gradients to suppress the water resonance [38]. Following eight dummy scans, each spectrum was acquired into 64 k datapoints over a spectral width of 20 ppm as the sum of 64 transients. The acquisition time was 2.73 seconds, giving a native free induction decay (FID) resolution of 0.183 Hz. The relaxation delay was set at two seconds. The total acquisition time was approximately six minutes per sample. An apodization function equivalent to a line-broadening of 0.3 Hz was applied to each FID prior to Fourier transformation. Assignment of peaks to specific metabolites was based on the addition of known standards to the biological samples, together with published literature [39], on-line metabolomics databases and statistical total correlation spectroscopy (STOCSY) [40]. Additional NMR spectroscopy experiments were conducted to assign a doublet at a chemical shift of δ 1.11. A one-dimensional ^1^H selective TOCSY experiment (See additional file 1, Figure S1) observed three related ^1^H resonances in the same molecule. Further two-dimensional experiments [See additional file 1, Figure S2] allowed the full ^1^H and ^13^C characterization [See additional file 1, Table S1] of the two diastereoisomers of 2,3-dihydroxybutanoic acid [See additional file 1, Figure S3]: 4-deoxy-erythronic acid and 4-deoxy-threonic acid, both of which have previously been identified in human urine [41].

### NMR data processing and statistical analysis

Data were imported and manipulated in Matlab (Mathworks, Natick, MA, USA) using in-house code for automatic phasing, baseline correction, and referencing chemical shifts to the TSP resonance at δ 0. To account for variance in dilution of the urine samples each spectrum was subsequently normalized by the median-fold change to a reference spectrum generated by calculation of the median of all spectra at each spectral point [42]. The spectra were analyzed at two resolutions: (1) reduced resolution of 0.01 ppm width (1,127 data points), for partial least squares (PLS) regression analysis; and (2) high resolution (32,697 data points), for covariance/correlation analysis.

For multivariate analysis the reduced resolution data were exported to SIMCA-P+ (Umetrics) with the TSP (δ < 0.15) and residual water (δ 4.675 to 4.895) resonances removed. PLS regression analysis was applied to these data to model optimally the association between the metabolic profile and the lifestyle and biological factors (U-Cd, age, sex, U-NAG and smoking status) and to identify individual metabolites significantly contributing to any associations observed. Permutation analysis was carried out for each PLS model to test for validity using 1,000 permutations per test [See additional file 1, Figure S4].

Additionally, we used a variable-wise covariance and correlation based analysis of the high resolution data to assist in the identification of individual metabolites associated with lifestyle/biological factors. The results were visualized by color-scale projection onto the plots to indicate the correlation of metabolites with the individual factors, with red indicating a high correlation and dark blue no correlation. The direction and magnitude of the signals represent the co-variation of the metabolites with the specific lifestyle factor.

We measured the area under selected spectral peaks for six specific metabolites (3-hydroxyisovalerate (3-HV), dimethylglycine (DMG), citrate, 4-deoxy-erythronic acid (4-DEA), creatinine and creatine) identified from the above analyses. These data were then analyzed by multiple linear regression (MLR) using SPSS (v19, IBM, Armonk, NY, USA) controlling for major confounding factors (age, sex and smoking status). We developed three separate MLR models for citrate. Model 1 excluded individuals who did not answer the question regarding smoking status (n = 3); model 2 additionally excluded all individuals classed as current smokers (n = 32); and model 3 additionally excluded current (n = 32) and past smokers (n = 35). Additionally, we used non-parametric tests (Kruskal-Wallis/Mann-Whitney or Spearman's ρ) to test for a significant (*P *< 0.05) difference or trend, respectively, in the concentration of citrate between the three classes of smoker (current-, past-, and never smoked). A normal distribution of values in each group in the dataset was observed (*P *< 0.05, Shapiro-Wilk test).

### Quantitation of the urinary biomarker 8-oxo-deoxyguanosine (8-oxodG)

Urinary-8-oxodG (U-8-oxodG) was quantified in samples from the upper and lower 15^th ^percentile according to U-Cd concentration, as described previously [43]. The U-8-oxodG concentrations were normalized to the creatinine concentration (taken from [37]) and a non-parametric Mann-Whitney test used to test for a significant (*P *< 0.05) difference between the groups. A non-normal distribution of values in the upper 15^th ^percentile group was observed (*P *< 0.05, Shapiro-Wilk test).

## Results

### Effects of Cd exposure on endogenous metabolism

**Figure 2:**
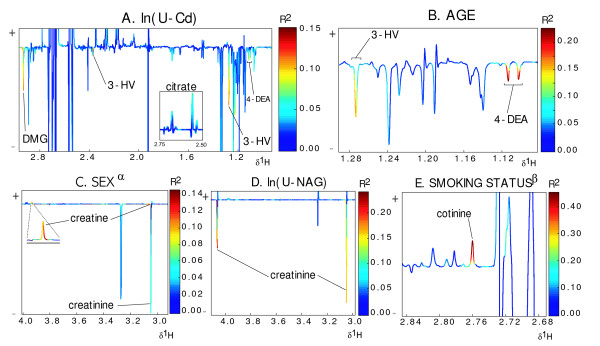
**Covariance and correlation between ^1^H NMR one dimensional urine spectra, and population characteristics**. The color code (R^2^) corresponds to the correlation coefficients of the variables with each of the characteristics: **A**, ln(U-Cd); **B**, age; **C**, sex; **D**, ln(U-NAG); and **E**, smoking status. N = 127. α - The direction and magnitude of the signals relate to the covariation of the metabolites with sex in the model, that is, female (positive) and male (negative). β - Smoking status was classified into two classes. 1 = never smoked (negative), 2 = current smoker (positive). 3-HV, 3-hydroxyisovalerate; DMG, dimethylglycine; 4-DEA, 4-deoxyerythronic acid. Spectra that exhibited signs of bacterial contamination, analgesics or ethanol were excluded from these analyses [See additional file 1 Table S2].

Our first objective was to determine if there were any associations between the global metabolic profile and U-Cd. Initial inspection of the spectral data indicated that a substantial number of individuals had detectable levels of analgesics (that is, paracetamol and ibuprofen) and/or ethanol detectable in their samples (51/178, approximately 29%, see additional file 1, Table S2). In order to avoid subsequent models being negatively affected by the global diuretic or pharmacological effects of these compounds on the spectra, these individuals were excluded from initial pattern recognition analyses. From the reduced resolution data-set (see Methods), PLS regression could predict U-Cd based on the metabolic profile data (Q^2 ^= 0.237, *P *< 0.01, Table 1). Inspection of model regression weights and bivariate analyses at both low and high resolution (Figure 2A) identified a number of urinary metabolites responsible for this association, namely: citrate, 3-HV, DMG, creatinine, creatine and 4-DEA. DMG, 3-HV, creatinine and 4-DEA were negatively correlated to U-Cd concentration and citrate was positively correlated.

**Table 1:** Statistics of multivariate analysis models demonstrating an association between ^1^H NMR spectroscopic data and several biological and lifestyle factors.

Model Description	Y variable	Number of LVs	R^2^X	Q^2^Y	Significance (*P *value)
**A**. PLS	ln(U-Cd)	3	0.251	0.237	< 0.01
**B**. PLS (current smokers excluded)	ln(U-Cd)	5	0.308	0.330	< 0.001
**C**. PLS (past and current smokers excluded)	ln(U-Cd)	1	0.0729	0.142	< 0.001
**D**. PLS	sex	3	0.241	0.104	> 0.05
**E**. PLS	age	2	0.216	0.224	< 0.001
**F**. PLS	ln(U-NAG)	1	0.054	0.162	< 0.001
**G**. PLS-DA	Smoking history^a^	2	0.194	0.185	< 0.01

^a^Smoking history was defined as either 1 = never smoked and past smoker (n = 106) or 2 = current smoker (n = 20), one individual did not complete the lifestyle questionnaire. Spectra that exhibited signs of bacterial contamination, analgesics or ethanol were excluded from these analyses. All variables were mean-centred and scaled to unit variance. NMR data were reduced to 1,127 data points of δ 0.01 resolution. Sample numbers for PLS models: A, D, E and F: n = 127. B: n = 106. C: n = 79. PLS-DA (model G) n = 126. Number of latent variables in a model were auto-fitted in SIMCA-P+. All models were assessed for validity by Y variable permutation analysis (1,000 permutations, see additional file 1 Figure S4). Scores scatter plots for each multivariate model can also be found in additional file 1 (Figure S5). ln(U-Cd), natural logarithm of urinary cadmium; ln(U-NAG), natural logarithm of urinary-N-acetyl-β-D-glucosaminidase; LV, latent variable; n, sample number; PLS, partial least squares; PLS-DA, partial least squares - discriminant analysis. R^2^X is the proportion of variance in the X matrix (i.e. spectral NMR data) described by the PLS model. Q^2^Y is the ability of the PLS model to predict the Y-score (ln(U-Cd), sex, age, ln(U-NAG) or smoking status) of a novel sample or the "cross-validated goodness-of-fit".

Since smoking is a major source of cadmium exposure we excluded smokers from PLS modelling to test if the link between metabolite profiles and U-Cd was independent of smoking status. PLS models with significant prediction of U-Cd could be generated either with current smokers excluded (Q^2 ^= 0.330, *P *< 0.001, Table 1) or with both current smokers and past smokers excluded (Q^2 ^= 0.142, *P *< 0.001, Table 1), suggesting that exposure to Cd from environmental and dietary sources alone may be sufficient to produce detectable metabolic effects.

### Association between other factors and endogenous metabolism

It has already been shown in this population that U-Cd is significantly associated with age, sex, U-NAG and smoking status [37]. In order to obtain a better understanding of possible causal links and the specificity of the responses to Cd we conducted further pattern recognition analyses to attempt to derive models for these potentially confounding factors. We could predict a statistically significant degree of variation by PLS regression in each of: age (Q^2 ^= 0.224, *P *< 0.001), smoking status (Q^2 ^= 0.185, *P *< 0.01) and U-NAG levels (Q^2 ^= 0.162, *P *< 0.001) from the urinary NMR data (Table 1). Inspection of regression weights for each of these models indicated that several metabolites correlated with U-Cd levels (that is, 3-HV, DMG, 4-DEA and creatinine) were also associated with these other physiological and lifestyle factors. Further specific metabolite correlates were defined, including cotinine to smoking status and creatine to sex (Figures 2B-2E).

### Regression analysis of individual urinary metabolite levels

Next we measured the area under selected spectral peaks (the integral) for the six metabolites (3-HV, DMG, citrate, creatinine, creatine and 4-DEA) identified by pattern recognition analysis as associated with U-Cd, and analyzed these in more detail by MLR. Samples previously excluded (n = 51) from pattern recognition analysis due to the presence of ethanol or analgesics were evenly distributed across age, smoking status, gender and U-Cd, reducing the likelihood that including these samples would confound the analysis [See additional file 1, Table S2]. Hence we felt it was justified to include these data for individual metabolite analyses to increase power wherever the relevant resonances could be integrated without interference. The integral of each metabolite was regressed as a function of U-Cd, age, sex and smoking status (Table 2). There was a significant association between citrate levels and both U-Cd (beta = 0.194, *P *< 0.05) and smoking status (beta = -0.298, *P *< 0.001) controlling for age and sex. Interestingly, citrate was positively correlated to U-Cd but negatively correlated to smoking status. This suggests that exposure to cigarette smoke via inhalation, although known to significantly increase Cd body burden, may have a different effect than Cd exposure alone via ingestion and/or inhalation (as assessed by U-Cd). Other metabolites showed significant associations to the potential confounding factors but not to U-Cd. 3-HV was negatively associated with age (beta = -0.326, *P *< 0.001) and sex (beta = -0.233, *P *< 0.01), and 4-DEA to age only (beta = -0.370, *P *< 0.001). Creatinine was negatively associated with age (beta = -0.427, *P *< 0.001) and sex (beta = -0.395, *P *< 0.001). Creatine was positively associated with sex only (beta = 0.321, *P *< 0.001). Again, we excluded smokers from the regression models, in order to establish if environmental exposure alone could account for the link between Cd body burden and perturbed endogenous metabolism. Citrate levels remained significantly associated with U-Cd while controlling for all other factors with either current smokers, or current and past smokers, removed from the regression model (Table 2; models citrate(2) and citrate(3) respectively).

**Table 2:** Correlation coefficients (standardized beta values) derived from multiple linear regression of key metabolite to lifestyle factors.

Metabolite	Chemical shift	Sample number	Correlation to Y variable			
	**(ppm)**	**number**	**(U-Cd)**	**age**	**sex**	**smoking status^a ^**

3-HV	1.276 to 1.269	160	-0.056	-0.326***	-0.233**	-0.023

DMG	2.936 to 2.920	176	-0.127	-0.122	-0.168	-0.052

citrate (1)	2.515 to 2.590	175	0.194*	-0.078	0.126	-0.298***

citrate (2)	2.515 to 2.590	143	0.228*	-0.060	0.157	-0.203*

citrate (3)	2.515 to 2.590	108	0.251*	-0.046	0.121	n/a

Creatinine	4.073 to 4.040	178	-0.010	-0.427***	-0.395***	0.015

Creatine	3.940 to 3.930	178	-0.006	0.088	0.321***	0.150

4-DEA	1.118 to 1.108	152	-0.042	-0.370***	-0.076	0.039

^a^Smoking status was classified into three classes. 1 = never smoked, 2 = past smoker and 3 = current smoker. Specific exclusions were made for each metabolite identified where coinciding interferences made the relevant resonance unsuitable for integration. All six metabolites were correlated to at least one of the epidemiological factors in either the PLS/PLS-DA regression analysis or the covariance/correlation models. Asterisks denote statistical significance (ANOVA with Bonferroni post-hoc correction): * *P *< 0.05, ** *P *< 0.01, *** *P *< 0.001. U-Cd was log normally distributed.

citrate (1) all three classes included (never smoked, past- and current-smoker). Note, three samples excluded. citrate (2) current-smokers additionally excluded. citrate (3) past- and current-smokers additionally excluded. ANOVA, analysis of variance; 3-HV, 3-hydroxyisovalerate; DMG, dimethylglycine; 4-DEA, 4-deoxyerythronic acid; PLS, partial least squares; PLS-DA, partial least squares - discriminant analysis; U-Cd, urinary cadmium.

**Figure 3:**
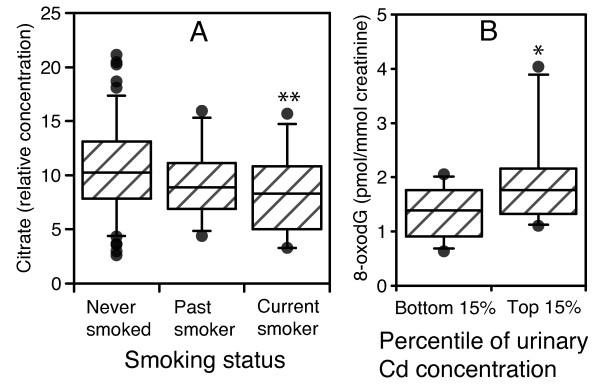
**The association between (A) relative citrate concentration and smoking status, and (B) U-Cd and U-8-oxodG**. (A) Normalized citrate levels in never, past and current smokers. ** statistically significant difference to 'never smoked' group, *P *< 0.01 (Kruskal-Wallis with Mann-Whitney post-test). Correlation coefficient (citrate versus smoking status), ρ = -0.225, *P *= 0.002 (Spearman's rho). Never smoked n = 108, past smoker n = 35, current smoker n = 32. (**B**) * statistically significant difference, *P *< 0.05 (Mann-Whitney test). N = 40, 20 per group. Median values marked with 95% confidence intervals represented as error bars. Data points falling outside these confidence intervals are marked.

Interestingly, there was a significant association between smoking status and citrate, even when current smokers were removed, suggesting that there was a metabolic difference between those individuals who had never smoked and those who had previously smoked. Normalized citrate levels were significantly different between groups (*P *< 0.05, Kruskal-Wallis test), specifically between current smokers and those who had never smoked (*P *< 0.01, Mann-Whitney test). There was also a clear negative trend in citrate levels with smoking status (Spearman's ρ = -0.225, *P *< 0.01), such that ex-smokers had intermediate levels of citrate on average compared to the other two groups (Figure 3A). This suggests that smoking could have induced a long-term change in citrate metabolism or transport, possibly reflecting an effect on mitochondria, which was manifested in decreased urinary citrate levels.

### Association of heavy metal exposure and oxidative stress

Alterations in citrate levels could imply changes in the consumption of citrate via the Krebs cycle in the mitochondrion. A perturbation to oxidative metabolism would be expected to lead to a change in ROS production and accompanying oxidative stress. Cd has been shown to increase ROS production *in vivo *[31,44] and indirectly damages DNA *in vitro *via oxidation by hydrogen peroxide production [45]. We attempted to define the association of heavy metal exposure (using U-Cd as an indicator) and the potential for oxidative DNA damage by measuring U-8-oxodG, which is widely used as a biomarker of oxidative stress [46,47]. We measured U-8-oxodG in a subset of 40 individuals (the upper and lower 15% of volunteers according to U-Cd concentration (Figure 3B)). The mean U-Cd concentration in the lower group was 0.115 ± 0.006 (nmol/mmol creatinine ± standard error of the mean (SEM)) and 0.766 ± 0.052 in the higher group. There was a significant (*P *< 0.05, Mann-Whitney test) increase in U-8-oxodG concentration (median ± SD) in the individuals in the upper 15^th ^percentile group (1.77 ± 0.82 pmol/mmol creatinine), compared to those in the lower group (1.39 ± 0.45 pmol/mmol creatinine). This suggests a positive association between U-8-oxodG and U-Cd, which could be representative of mitochondrial dysfunction and increased ROS production, consistent with our hypothesis.

## Discussion

Mechanistic evidence of chemical toxicity is vital to allow accurate risk assessment; in this context, multiple intermediate biomarkers may serve as combinatorial signatures of early molecular events of toxicity. Metabolic phenotypes can be used as an information-rich endpoint in molecular epidemiology and can define the interactions of lifestyle, environment and genes that determine diseases [10]. We have shown here that metabolic profiling can be successfully applied to a human toxicological/molecular epidemiological study, specifically in a population exposed to Cd pollution derived through environmental exposure, to define associations between the metabolome, lifestyle and exposure data, and other molecular biomarkers of tissue damage.

U-Cd concentration in the majority of individuals living at the Avonmouth site was within typical estimated levels of a normal population (0.1 to 0.6 nmol/mmol creatinine [16]), and only 25% of individuals had U-Cd levels above 0.5 nmol/mmol creatinine [37] which is the lower limit of potential risk of renal damage suggested by the Scientific Committee on Toxicity, Ecotoxicity and the Environment [48] (CSTEE). The joint FAO/WHO expert committee on food additives (JECFA) concluded that an excess prevalence of tubular renal dysfunction would not be expected below a U-Cd level of 2.5 nmol/mmol creatinine [49], considerably higher than the range of U-Cd concentrations at which we observed metabolic changes. An NMR-based metabolic profiling approach could complement exposure assessment as it details actual biochemical disturbance and could aid the determination of the mechanism of action of a chemical toxicant and the etiology of associated disease.

We identified a previously unreported association of urinary citrate to both Cd exposure and smoking. Surprisingly, citrate was positively correlated with U-Cd and negatively with smoking status, despite smoking being an important source of cadmium exposure. The increase in urinary citrate levels associated with U-Cd may result from Cd-induced tubule toxicity that affects citrate handling in the kidney [50], although we did not observe a direct association between citrate levels and tubular dysfunction (as represented by U-NAG levels). Smoking may reduce urinary citrate levels due to reduced capacity for oxidative metabolism in mitochondria. Chronic smoking is associated with a decrease of complex IV and III activity in the lymphocyte mitochondrial electron transfer chain, which returns to normal after cessation of smoking [51,52]. There was also a positive association between U-Cd and U-8-oxodG, which is indicative of increased systemic oxidative stress and may also result from mitochondrial dysfunction. Past smokers also had intermediate urinary citrate levels relative to current smokers and those who had never smoked. This observation suggests that it may be possible to monitor biological effects of exposure in individuals who are no longer exposed to the causative agent, whether they be irreversible or slow to return to a 'normal' metabolic phenotype.

Using a non-targeted approach 3-HV was found to be negatively correlated to U-Cd, age and sex, and DMG negatively correlated to U-Cd and sex. However, after allowing for confounding factors it was not conclusively shown that these two metabolites were associated with U-Cd, possibly due to the limitation of sample numbers. Despite this, correspondence between our observations and other studies of the biochemical effects of Cd suggest that these metabolic changes may still be related to the underlying mechanisms of Cd toxicity. Cd inhibits oxidoreductases involved in vitamin D metabolism [53] and 3-HV is a product of the L-leucine mitochondrial catabolic pathway which also involves this class of enzymes. One hypothesis is that reduction in urinary 3-HV could be occurring in parallel to direct mitochondrial damage [31,54]. Another of the six metabolites identified as correlated to U-Cd, 4-DEA, is also involved in amino acid catabolism: it is a breakdown product of threonine. Threonine deaminase converts threonine to alpha-ketobutyrate, a precursor of isoleucine, and this is further modified by reductases to 4-deoxyerythronic acid and its diastereoisomer 4-deoxythreonic acid [55] (also observed in our study but not correlated to U-Cd).

Our study had several limitations: clearly our observations need to be replicated in other larger cohorts and we did not have available repeated or alternative biological specimens from the study participants. Metabolite measurements in blood plasma for instance would provide clarification as to whether the observed associations were systemic in origin or likely to reflect mainly renal effects. A detailed analysis of the contribution of diet and activity to specific metabolite variation was also not possible. These issues are currently being examined in follow-up studies (for example, http://www.envirogenomarkers.net). In particular it will be important to evaluate the additional prognostic value of metabolomic measurements compared to established clinical pathology data and exposure assessment if the metabolites observed here associated with Cd burden are to be considered as valuable biomarkers in the future.

## Conclusions

Using an NMR-based approach, we have demonstrated the capacity, in principle, of metabolic profiling to characterize the metabolic consequences of exposure to environmental toxicants, such as Cd and tobacco smoke, in a human population. We conclude that metabolic profiling has the potential to identify novel biomarkers and molecular signatures of the effects of exposure to many environmental toxicants, and thus improve risk assessment models, ultimately guiding intervention to prevent disease progression. As such, metabolic profiling represents a vital element in future initiatives to define the human exposome. In the longer term, the methods employed in the present Cd exposure study could be used on a broad scale for baseline surveys in health impact assessments for development projects, going on to play a central role in monitoring populations living near point sources of pollution such as industrial sites.

## Abbreviations

3-HV: 3-hydroxyisovalerate; 4-DEA: 4-deoxyerythronic acid; 8-oxodG: 8-oxo-deoxyguanosine; CSTEE: Scientific Committee on Toxicity, Ecotoxicity and the Environment; DMG: dimethylglycine; FAO: Food and Agriculture Organization; FID: free induction decay; JECFA: Joint FAO/WHO Expert Committee on Food Additives; MLR: multiple linear regression; NAG: N-acetyl-β-D-glucosaminidase; NMR: nuclear magnetic resonance; PLS: partial least squares; PLS-DA: PLS-discriminant analysis; ROS: reactive oxygen species; SEM: standard error of the mean; TSP: 3-trimethylsilyl-1-[2, 2, 3, 3, -^2^H_4_] propionate; U-Cd: urinary-Cd; U-NAG: urinary-NAG.

## Competing interests

The authors declare that they have no competing interests.

## Authors' contributions

HCK, JGB, LJ, CS, DJS and LDKT designed the experiment. JKE, TJA, MPT, JGB and HCK performed the metabonomic experiments, analyzed data and wrote the research article. LDKT and LJ also contributed to the experimental work and the writing of the article. FT and RS performed the 8-oxodG assay and analyzed the associated data. All authors listed here were involved in drafting the manuscript and they have read and approved the final version for publication.

## Author information

The late Lars Järup MD, PhD was involved in initiating the study and his contributions were instrumental to its success.

## Pre-publication history

The pre-publication history for this paper can be accessed here:

http://www.biomedcentral.com/1741-7015/10/61/prepub

## Supplementary Material

Additional file 1 **Supporting information for metabolite identification and PLS model validity**. The additional file includes NMR spectroscopic data that were acquired to elucidate the structure of an unknown urinary metabolite, details of samples excluded from the multivariate analysis and validation and scores plots of the PLS models. Click here for file
