# Supplementary material for: Metabolic profiling detects early effects of environmental and lifestyle exposure to cadmium in a human population
Source: BMC Med. 2012 Jun 19;10:61. doi: 10.1186/1741-7015-10-61 (PMC3391181; doi:10.1186/1741-7015-10-61)
Supplement: Additional file 1 — Supporting information for metabolite identification and PLS model validity. The additional file includes NMR spectroscopic data that were acquired to elucidate the structure of an unknown urinary metabolite, details of samples excluded from the multivariate analysis and validation and scores plots of the PLS models. [file 1741-7015-10-61-S1.DOC]

**Additional file 1**

**Metabolic profiling detects early effects of environmental and lifestyle exposure to cadmium in a human population.**

James K Ellis, Toby J Athersuch, Laura D K Thomas, Friederike Teichert, Miriam Pérez-Trujillo, Claus Svendsen, David J. Spurgeon, Rajinder Singh, Lars Järup, Jacob G Bundy and Hector C Keun.

The identification and complete ^1^H and ^13^C NMR characterization of the unknown metabolite (doublet at 1.11ppm (**a**)) was achieved by means of 1D selective and 2D NMR experiments. The 1D ^1^H selective TOCSY experiment shows a multiplet **b** (doublet of quartets) at 4.11 ppm and a doublet **c** at 4.08 ppm when the signal of interest (**a)** was irradiated (Figure S1).


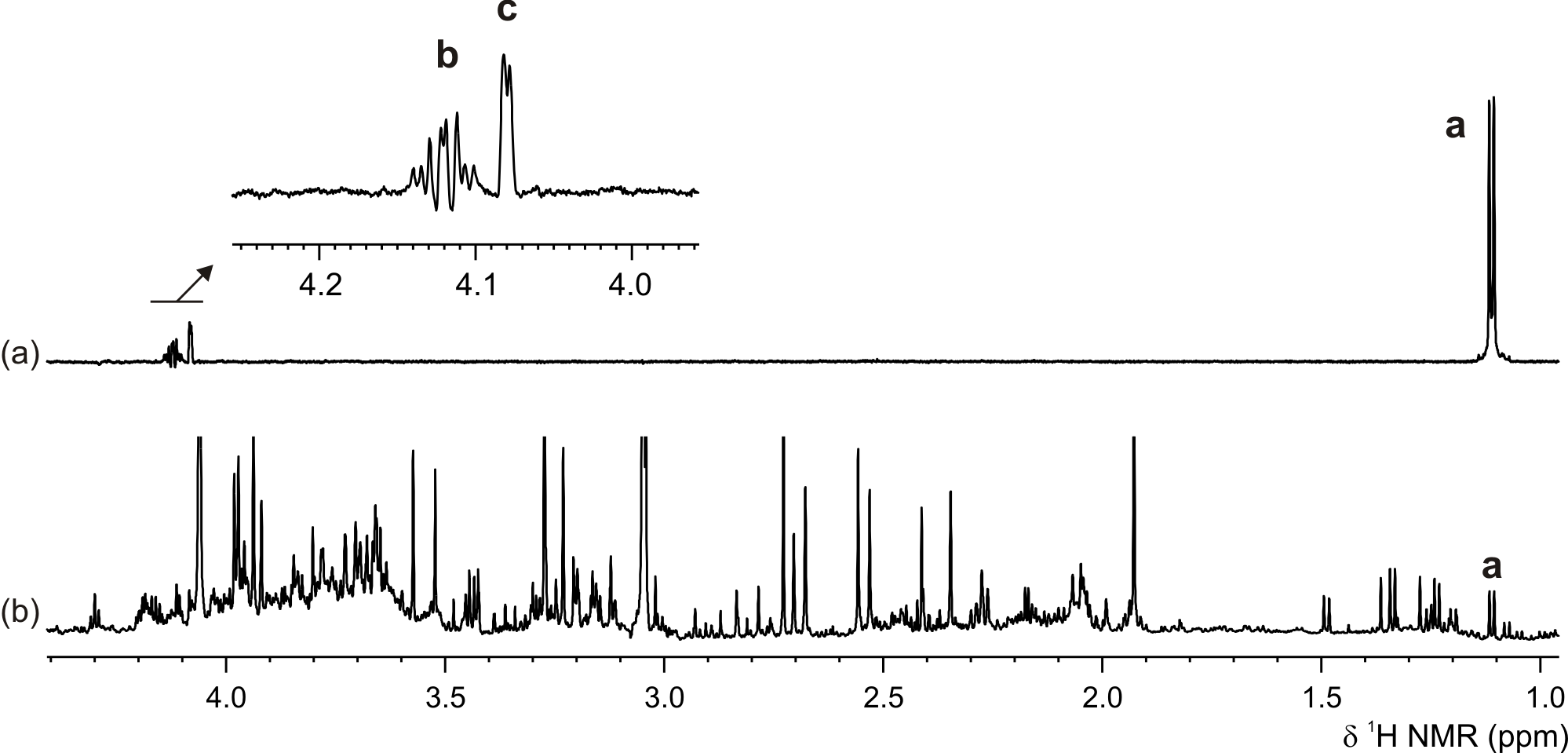


Figure S1. 1D ^1^H NMR spectra of human urine sample (a) 1D ^1^H selective TOCSY with solvent suppression and irradiation of the doublet at 1.11 ppm. (b) ^1^H NMR spectrum with solvent suppression.

The ^1^H-^13^C HSQC (Table S and Figure S2) experiment allowed the identification of the carbons directly bonded to the protons in the unknown metabolite. Carbons **b** and **c** (Figure S3) resonate at 71.7 and 78.6 ppm, respectively, which correspond to typical chemical shifts of carbons directly bonded to a hydroxyl group, while carbon **a** resonate at 18.6 ppm. The results of the ^1^H-^13^C HMBC experiment confirmed the final structure of the unknown metabolite (1.11ppm) as the diastereoisomer of 2,3-dihydroxybutanoic acid: 4-deoxyerythronic acid. The previous assignments were confirmed and a coupling between proton **c** and a carboxylic carbon (**d**) at 180.3 ppm was observed (Figure S2, Figure S3 and Table S1). Both diastereoisomers of 2,3-dihydroxybutanoic acid were identified in samples in the current study.


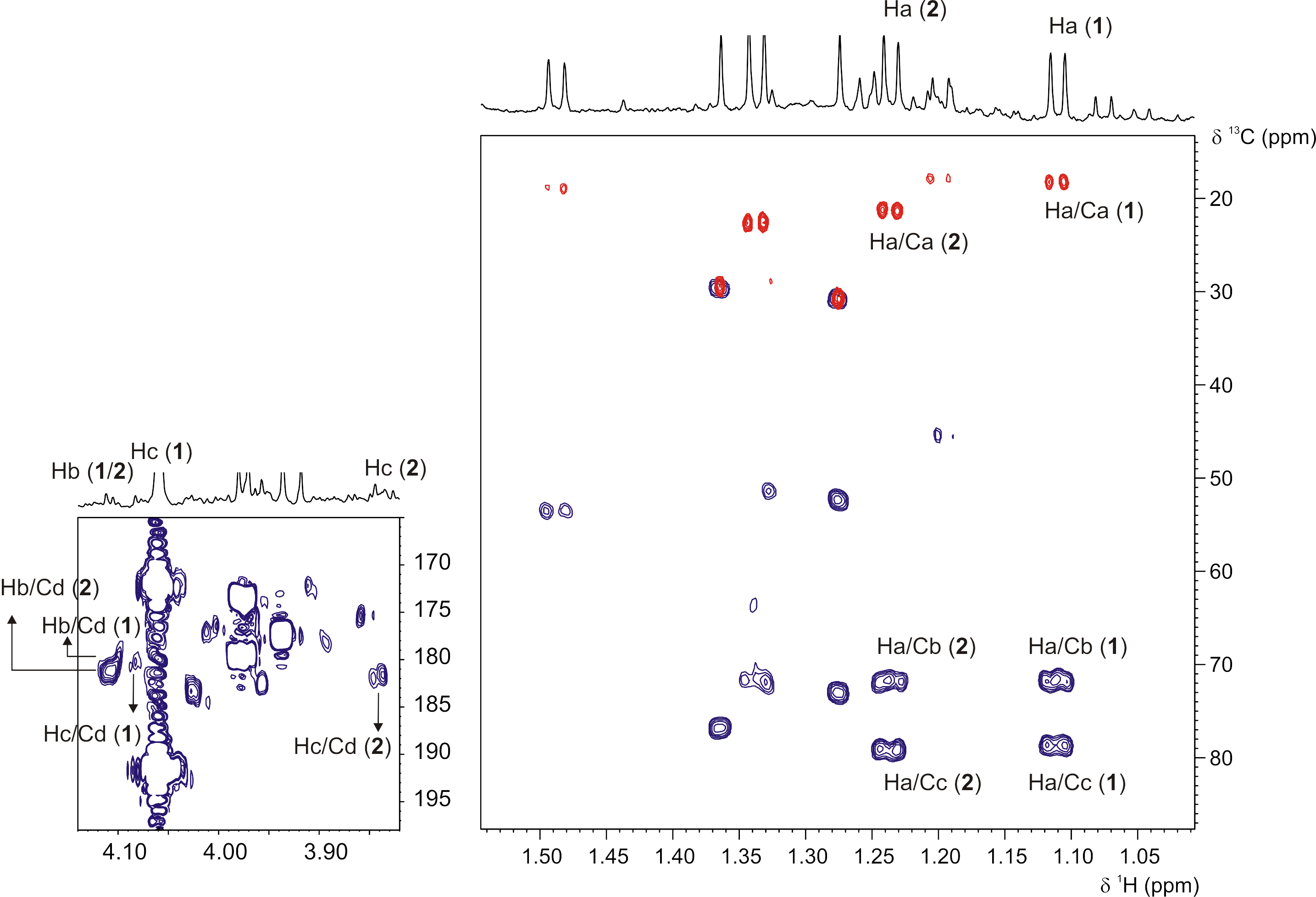


Figure S2. Expanded regions of ^1^H-^13^C HSQC (cross peaks in red) and ^1^H-^13^C HMBC (cross peaks in blue) experiments.


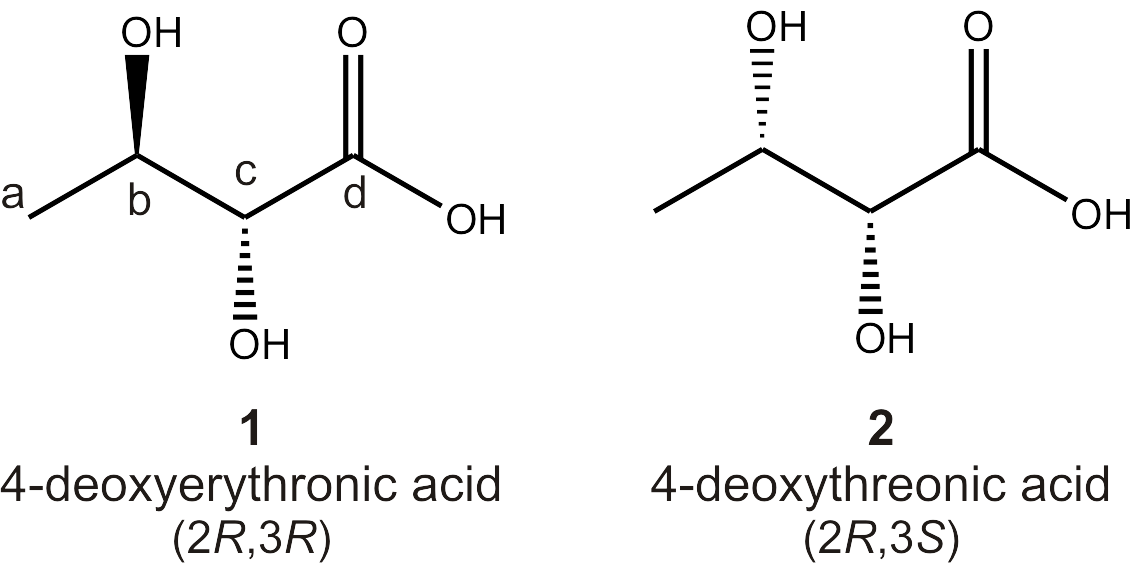


Figure S3. Diastereoisomers of 2,3-dihydroxybutanoic acid: 4-deoxyerythronic acid (**1**) and 4-deoxythreonic acid (**2**).

Table S1. Description of ^1^H and ^13^C NMR chemical shifts (δ) and H,H-coupling constants (^3^*J*_H,H_) of compounds **1** (4-deoxyerythronic acid) and **2** (4-deoxythreonic acid (Figure S3.)).

| Compound 1  4-deoxyerythronic acid | | | Compound 2  4-deoxythreonic acid | |
| --- | --- | --- | --- | --- |
|  | δ(^1^H) [ppm] and ^3^*J* _H,H_ [Hz] | δ(^13^C) [ppm] | δ(^1^H) [ppm] and  ^3^*J* _H,H_ [Hz] | δ(^13^C) [ppm] |
| **d** | - | 180.4 | - | 181.7 |
| **c** | 4.079 (d) | 78.6 | 3.843 (d) | 79.1 |
| **b** | 4.112 (m) | 71.7 | 4.115 (m) | 71.7 |
| **a** | 1.110 (d, *J*=6.4) | 18.6 | 1.235 (d,*J*=6.4) | 21.6 |

Table S2. The mean age and urinary cadmium concentration in the sub-set of samples removed (n=51) and used (n=127) for multivariate analysis.

SEM= Standard Error of the Mean. crn=creatinine.

|  | Sample set (number of samples) | | | | | | | |
| --- | --- | --- | --- | --- | --- | --- | --- | --- |
|  | 127 | | | | 51 | | | |
|  | Mean | SEM | Min | Max | Mean | SEM | Min | Max |
| Age | 52.3 | 1.5 | 18 | 86 | 58.1 | 1.7 | 34 | 81 |
| U-Cd (nmol/mmol crn) | 0.35 | 0.02 | 0.07 | 1.44 | 0.39 | 0.06 | 0.08 | 2.33 |

The sample set removed (n=51) from the multivariate analysis was comprised of 17 male and 34 female participants, with 29 non-smokers (never smoked), 8 past-smokers AND 12 currents-smokers (two did not answer). For comparison, the sample set included (n=127) in the multivariate analysis was comprised of 57 male and 70 female participants, with 79 non-smokers (never smoked), 27 past-smokers and 20 current-smokers (one did not answer).

crn = creatinine.


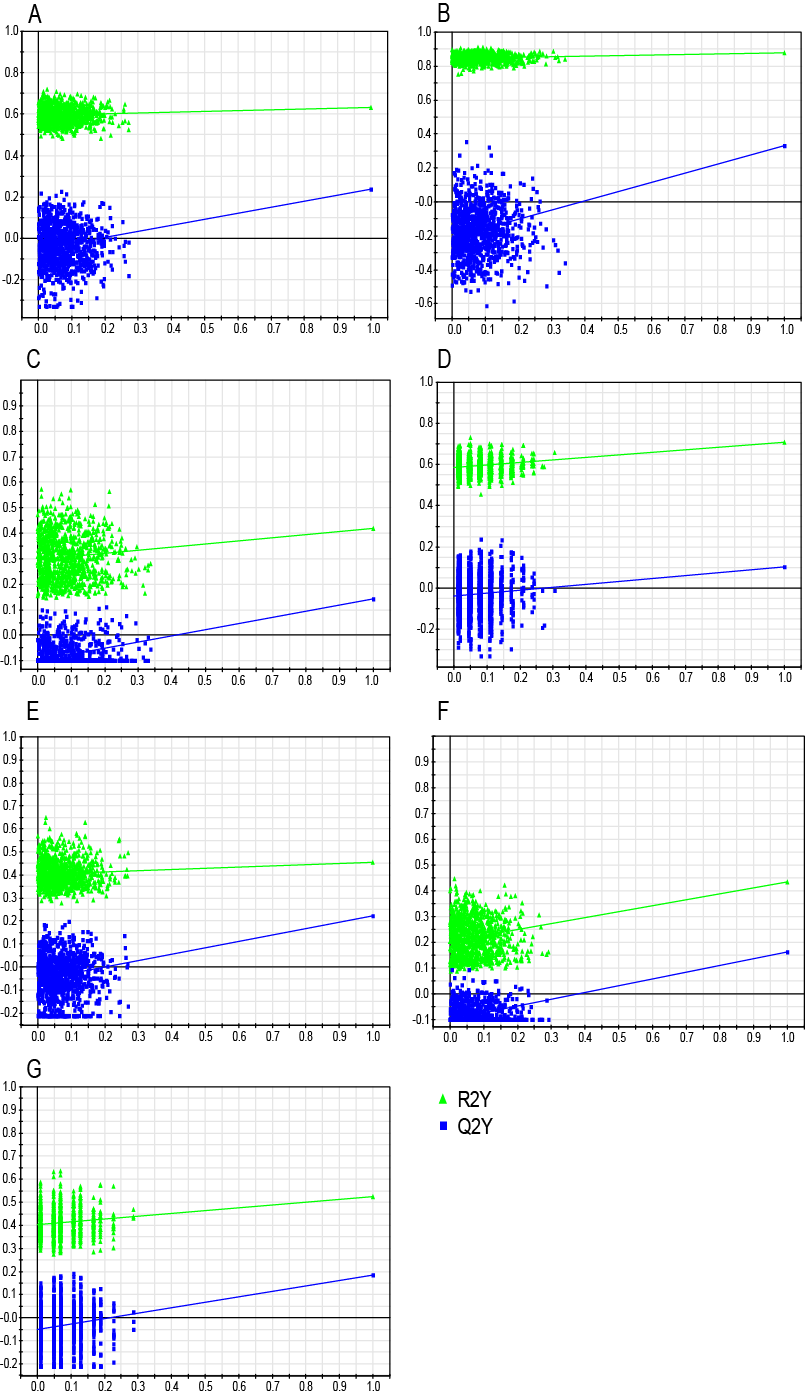


Figure S4. Validation plots of each PLS model in Table 1. Permutation analysis was conducted for each PLS model to test for validity (1000 permutations per test).

Y variables for each PLS model (full description of models can be found in Table 1 of manuscript) are as follows: A=ln(U-Cd), B=ln(U-Cd), C=ln(U-Cd), D=sex, E=age, F=ln(U-NAG) and G=smoking status. A *p* value was obtained by assessing the distribution of the original Q2Y classification in all the permuted values, as previously described [1, 2].


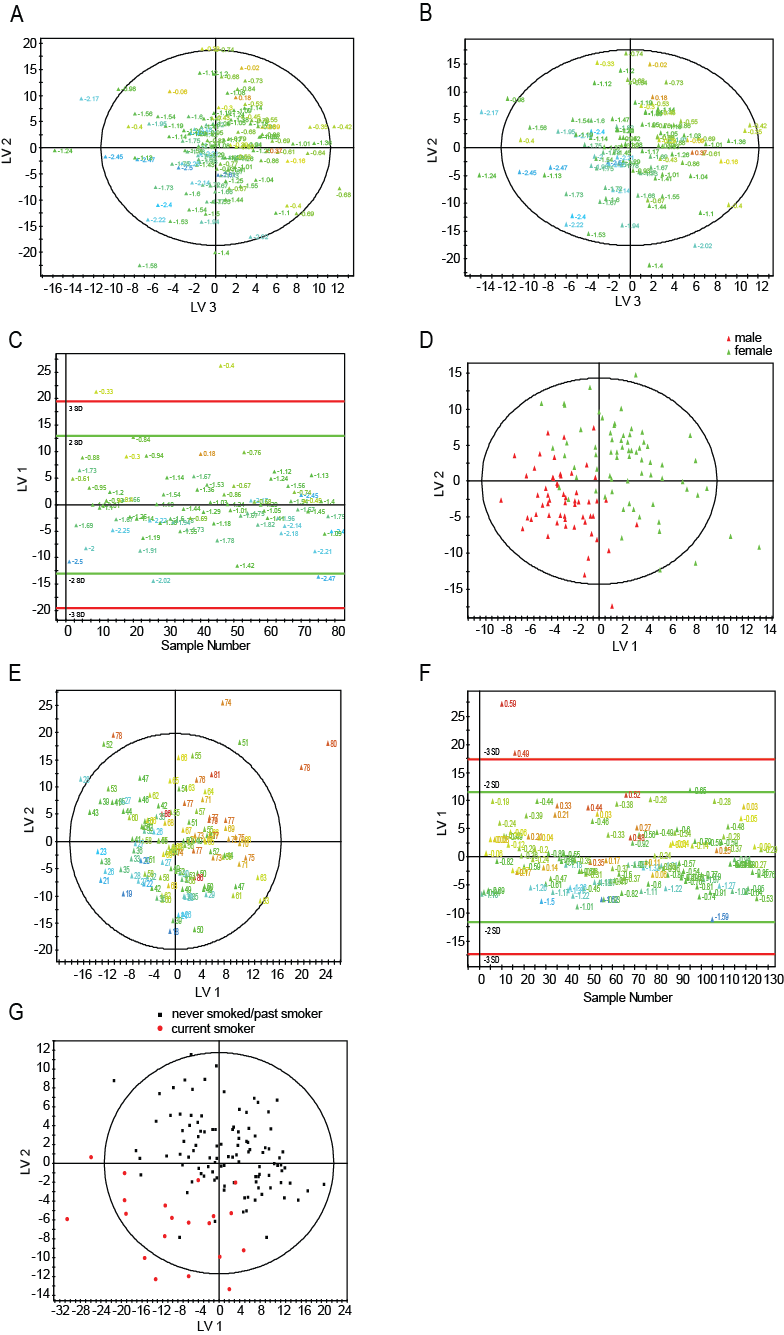


Figure S5. Scores scatter plot for each PLS model in Table 1. All variables were mean-centred and scaled to unit variance. NMR data were reduced to 1127 data points of δ 0.01 resolution. Sample numbers for PLS models: A, D, E & F; n=127. B; n=106. C; n=79. PLS-DA (model G) n=126. LV: Latent Variable. Number of latent variables in model were auto-fitted in SIMCA-P+. All models were assessed for validity by Y variable permutation analysis in SIMCA-P+ (1000 permutations, see Figure S4). Models A-C are coloured and labelled by ln(U-Cd). Model D and G are coloured by class (gender and smoking respectively). Model E and F are coloured and labelled by age and ln(U-NAG) respectively. Model G: Smoking history was defined as either 1=never smoked or past smoker (n=106), 2=current smoker (n=20), one individual did not complete lifestyle questionnaire.

**Reference List**

1. Westerhuis JA, Hoefsloot HCJ, Smit S, Vis DJ, Smilde AK, Velzen EJJ, Duijnhoven JPM, Dorsten FA: **Assessment of PLSDA cross validation**. *Metabolomics* 2008, **4**:81–89.

2. Backshall A, Alferez D, Teichert F, Wilson ID, Wilkinson RW, Goodlad RA, Keun HC: **Detection of metabolic alterations in non-tumor gastrointestinal tissue of the Apc(Min/+) mouse by (1)H MAS NMR spectroscopy.** *J. Proteome Res.* 2009, **8**:1423–1430.
